# *Plasmodium falciparum* histidine rich protein 2 (*pfhrp2*): an additional genetic marker suitable for anti-malarial drug efficacy trials

**DOI:** 10.1186/s12936-021-04014-4

**Published:** 2022-01-04

**Authors:** Wahib M. Atroosh, Yee-Ling Lau, Georges Snounou, Meram Azzani, Hesham M. Al-Mekhlafi

**Affiliations:** 1 Department of Parasitology, Faculty of Medicine, Universiti Malaya, 50603 Kuala Lumpur, Malaysia; 2 Department of Microbiology and Parasitology, Faculty of Medicine and Health Sciences, University of Aden, Aden, Yemen; 3 CEA-Université Paris Sud 11-INSERM U1184, Immunology of Viral Infections and Autoimmune Diseases (IMVA-HB), IDMIT Department, IBFJ, DRF, Fontenay-aux-Roses, France; 4 Department of Community Medicine, Faculty of Medicine, MAHSA University, Bandar Saujana Putra, Selangor Malaysia; 5 Department of Parasitology, Faculty of Medicine and Health Sciences, Sana’a University, Sana’a, Yemen; 6 Medical Research Centre, Jazan University, Jazan, Kingdom of Saudi Arabia

**Keywords:** Malaria, *Plasmodium falciparum*, Drug efficacy clinical trial, *hrp2*, *msp1*, *msp2*, *Glurp*

## Abstract

**Background:**

Genotyping of the three *Plasmodium falciparum* polymorphic genes, *msp1*, *msp2* and *glurp*, has been adopted as a standard strategy to distinguish recrudescence from new infection in drug efficacy clinical trials. However, the suitability of a particular gene is compromised in areas where its allelic variants distribution is significantly skewed, a phenomenon that might occur in isolated parasite populations or in areas of very low transmission. Moreover, observation of amplification bias has diminished the value of *glurp* as a marker.

**Methods:**

The suitability of the polymorphic *P. falciparum* histidine-rich protein 2 (*pfhrp2*) gene was assessed to serve as an alternative marker using a PCR-sequencing or a PCR–RFLP protocol for genotyping of samples in drug efficacy clinical trials. The value of *pfhrp2* was validated by side-by-side analyses of 5 admission-recrudescence sample pairs from Yemeni malaria patients.

**Results:**

The outcome of the single *pfhrp2* gene discrimination analysis has been found consistent with *msp1*, *msp2* and *glurp* pool genotyping analysis for the differentiation of recrudescence from new infection.

**Conclusion:**

The findings suggest that under the appropriate circumstances, *pfhrp2* can serve as an additional molecular marker for monitoring anti-malarials efficacy. However, its use is restricted to endemic areas where only a minority of *P. falciparum* parasites lack the *pfhrp2* gene.

**Supplementary Information:**

The online version contains supplementary material available at 10.1186/s12936-021-04014-4.

## Background

Malaria drug efficacy clinical trials are conducted to monitor the continued efficacy of front-line anti-malarial drugs, particularly against falciparum malaria. After administration of full-dose malaria treatment, in vivo drug efficacy is assessed through clinical and parasitological follow-up according to standard protocols [1]. The erstwhile recommended follow-up duration of 14 days was revised to a standard 28 days that can be extended to 42 days or to 63 days for the drugs with a long half-life [1, 2]. Given the short incubation period of *P. falciparum*, longer follow-up periods increase the likelihood of acquiring new infections, especially in areas of high malaria transmission, which can lead to an underestimate of drug efficacy through mistaken classification of newly acquired infections as drug failures. In order to counter this, molecular genotyping strategies based on polymerase chain reaction (PCR) amplification have been developed to correct estimates of drug efficacy in clinical trials by enabling a distinction between recrudescences versus new infections during the follow-up [3]. The genetic markers recommended by the World Health Organization (WHO) for PCR correction are the polymorphic regions of three *P. falciparum* genes: merozoite surface protein 1 (*msp1*, block2), merozoite surface protein 2 (*msp2*, block3), and the repeat region 2 of the glutamate-rich protein (*glurp*) [4]. The amplified allelic variants are classified according to size, and also to allelic type for the *msp1* and *msp2* [4].

The suitability of each marker depends on the local parasite population to comprise numerous allelic variants with an unbiased distribution. This is likely in areas of high transmission intensity where parasites are genetically diverse and infections are often polyclonal, with a multiplicity of infection (MOI) often reaching 5, but less so in areas of low transmission with low MOI (1–2), where re-infections by the same genotype might occur more frequently. Furthermore, recent investigations showed that *glurp* genotyping is affected by amplification bias, thereby diminishing confidence in this marker for PCR correction strategies [5, 6]. Reliance on two genetic markers, as compared to three, will diminish the accuracy of PCR correction, and might lead to an overestimation of drug failures, which could prompt an unnecessary change of the first-line treatment, that is advocated when PCR-corrected failure rates exceed 10% [1, 6, 7].

Defined polymorphic microsatellite markers, that vary by the number of repeated short nucleotide sequences, have been used as an alternative or as an additional standard genotyping marker [8–11]. Microsatellite markers are usually located in non-coding regions and are considered to be selectively neutral. However, they are known for their instability, with the frequency of repeats within some loci capable of changing during the infection [2, 10], which might lead to underestimate drug failure rates [2, 10, 12]. Moreover, microsatellite markers require a range of 6–13 PCR reactions, based on the number of loci included in the analysis [8, 13, 14] (Table 1).

**Table 1:** Comparison of *pfhrp2* genotyping protocol with those of other markers generally used in drug efficacy trial

Protocol difference	*pfhrp2*	*msp1* + *msp2*	Microsatellites
Molecular protocol	Single-run PCR or PCR–RFLP	Nested PCR	Nested PCR
Sequencing	Yes**	No	No
Restriction enzyme	Yes***	No	No
No. of PCR reaction	1	7	6*
Number of primers	2	14	10–12*

*Minimum for 5 loci, **For PCR-Sequencing protocol option, ***For PCR–RFLP protocol option

The suitability of another polymorphic gene, coding for the *P. falciparum* histidine-rich protein 2 (*pfhrp2*), was assessed as an additional marker for the discrimination between recrudescent and new infections. Two genotyping protocols were designed and evaluated, PCR amplification followed by sequencing, and PCR followed by restriction enzyme length polymorphism (PCR-RELP) analysis.

## Methods

### Study setting

In an in vivo study that aimed to assess the efficacy of artemisinin + sulfadoxine-pyrimethamine (AS-SP) drug combination against uncomplicated falciparum malaria in the Tehama region of Yemen [15], recurrent parasitaemia during follow-up was detected in five cases. Of these cases, three were classified as true recrudescence as they were found to carry the same malaria genotypes using the *msp1*, *msp2* and *glurp* markers. During this study, the impact of *pfhrp2* gene variation on the performance of *hrp2*-based RDT was evaluated in this area. Sequencing analysis revealed high genetic diversity for *pfhrp2* with notably low allelic variant frequency distribution [16].

### Malaria isolates

Five archived filter paper blood spot admission (prior to treatment baseline, or day 0) and recurrence (day X after ACT administration) sample pairs of *P. falciparum* infection were included in this analysis. During the 28 days of follow-up: one case recurred at day 14, two at day 21 and two others at day 28.

### DNA extraction

Two to three discs (6 mm diameter) of the collected filter paper blood spot were utilized for genomic DNA extraction using Qiagen blood and tissue kit (QIAGEN, DNeasy^®^ Blood & Tissue Kit, Germany). The DNA was eluted in 35 µL AE elution buffer (10 mM Tris–Cl; 0.5 mM EDTA; pH 9.0) and stored at −20 °C until use.

### PCR amplification and sequencing analysis of pfhrp2

Amplification of *pfhrp2* was carried out in a single-run PCR using a specifically designed oligonucleotide primer pair (PfHRP2-F: 5′-TGTGTAGCAAAAATGCAAAAGG-3′ and PfHRP2-R: 5′ TTAATGGCGTAGGCAATGTG-3′) flanking the polymorphic exon-2 region of the *pfhrp2* gene, as previously described [16]. Briefly, 3 µL of the genomic DNA was added to total up 50 µL reaction volume containing 20 µL ExPrime Taq Premix (Genet Bio, Korea) and 2 µL of each forward and reverse primers (10 µM). Thermal cycling condition was initiated with a denaturation cycle at 95 °C for 5 min followed by 40 cycles (denaturation step at 95 °C for 30 s, annealing at 57 °C for 40 s and an extension step at 72 °C for 90 s) and a final extension cycle at 72 °C for 10 min. All amplification reactions were performed using MyCycler™ or T100 Thermal Cyclers (BioRad, Hercules, USA). The PCR products were visualized in 2% agarose gel pre-stained with Sybr^®^ safe DNA gel stain (Invitrogen, USA) using a gel-documenting system (Bio-Rad, Hercules, CA, USA). Amplicon lengths in base pair were reported against 100 DNA ladder [16].

The PCR product bands were cut out of the gels, purified, and sequenced in both directions. The corresponding consensus amino acid sequences were aligned using BioEdit Sequence Alignment Editor Software (version 7.1.9) and Molecular Evolutionary Genetics Analysis (Mega) software (version 7.0.26) and analysed for the type and number of amino acid repeat units [16]. The sequences for the sample pairs (day 0 sample and day X) from this study were aligned and analysed for similarity in the type and number of the amino acid repeat units. A day X sample was classified as true recrudescent (treatment failure) if it showed 100% similarity in the amino acid repeat type and number as compared to that from the day 0 baseline sample. A difference in the type and/or in the number of one or more of the amino acid repeat units led the sample to be classed as a new infection.

### PCR–RFLP analysis of pfhrp2

Given that sequencing might not be readily available in some settings, a genotyping protocol based on restriction enzyme length polymorphism (RFLP) analysis of the amplified fragment was designed and evaluated. Each PCR product was subjected to digestion using one of two restriction enzymes: *Alu* I (cutting site AGCT) and *Pv*u II (cutting site CAGCTG). Post-digestion products were then analysed in 2.5% agarose gels and the number and size of fragments reported for each sample. Identical digestion patterns between day 0 and day X paired samples indicated a treatment failure, while a difference indicated a new infection.

### msp1, msp2 and glurp genotyping

Amplification and analyses of *msp1*, *msp2* and *glurp* genes were performed using nested PCR according to previously described standard protocols [3, 4, 17]. Amplicons were visualized in 2.5% agarose gel stained with Sybr^®^ safe DNA gel stain (Invitrogen, USA) using UV documenting system (Bio-Rad, Hercules, CA, USA). The size of the PCR products for each marker was reported and then grouped into different size bins of 25 bp for *msp1* and *msp2*, and 50 bp for *glurp*. For the recurrent parasitaemia during the in vivo follow-up period, samples of both day 0 (i.e., pre-treatment) and their corresponding samples collected at day X (i.e. post-treatment) were genotyped and compared for alleles similarities.

### Determining allelic frequency distribution for pfhrp2

The type and frequency of the *pfhrp2* alleles in the study area were derived using the *pfhrp2* sequence of 180 samples obtained from the previous study [16]. A difference of one amino acid or more was considered sufficient to define a new allelic type, and allelic frequencies were then calculated.

### Determining allelic frequency distributions for msp1, msp2 and glurp

At least fifty samples are recommended to estimate the allelic frequency and the multiplicity of infection (MOI) of the *msp1*, *msp2* allelic families and *glurp* genotypes [1]. Thus, fifty archived blood samples of *P. falciparum* mono-infection were randomly selected and genotyped as previously described [17]. A representative size fragment for each *msp1* allelic family (K1, MAD 20 and RO33) and *msp2* allelic family (3D7/IC and FC27), as well as *glurp* allelic variants, were subjected to sequencing to confirm the nature of the allelic variants.

## Results

### Distribution of msp1, msp2 and glurp alleles

**Fig. 1:**
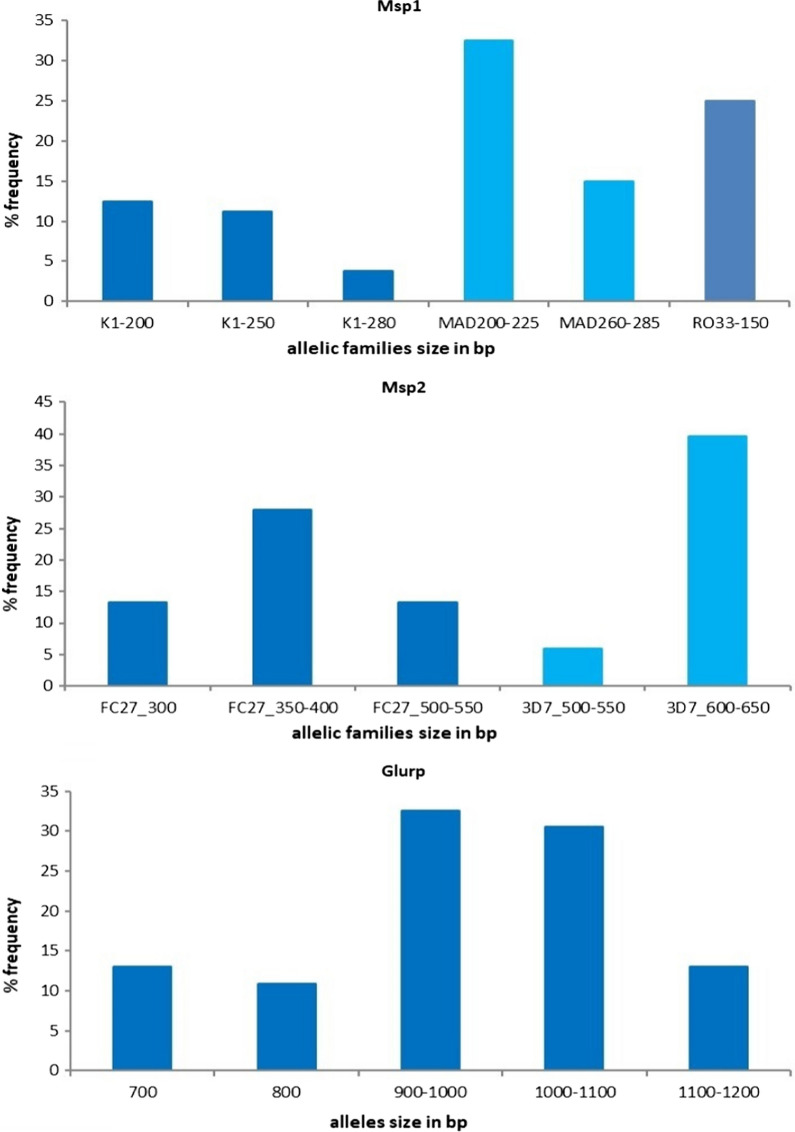
Distribution of the *P. falciparum msp1*, *msp2* and *glurp* markers allelic variants in 46 isolates from the Tehama region, Yemen

Out of the fifty randomly selected samples, 46 were successfully amplified for all three markers: *msp1*, *msp2* and *glurp*. Six allelic variants were observed for *msp1*, five for the *msp2* and five for *glurp*. The frequencies of the 200–225 bp MAD20, and 150 bp RO33 *msp1* allelic variants, and the 350–400 bp FC27 and 600–650 bp 3D7/IC *msp2* allelic variants were biased as they were found in 32.5%, 25%, 28% and 39.7% of the examined samples, respectively. Similarly, the *glurp* alleles 900–1000 and 1000–1100 bp allelic variants were predominant with frequencies of 32.6%, and 30.5%, respectively. Therefore, distinguishing recrudescence from new infection in this area using these alleles might be challenging. Nonetheless, the other 10 allelic variants of *msp1*, *msp2* and *glurp* had low frequencies (3.8–15%) (Fig. 1). The MOI of the *P. falciparum* isolates analysed was 1.7, 1.5 and 1.0 for *msp1*, *msp2* and *glurp*, respectively. Thus, the validity of these three markers for PCR correction was sub-optimal, and likely to over-estimate treatment failures.

### Diversity and frequency distribution of the pfhrp2 alleles

In a previous survey of *pfhrp2* gene variation [16] conducted in the same area, all isolates (189) were successfully amplified (100% amplification rate) and the *pfhrp2* fragment obtained successfully sequenced. The isolates were all found to carry a single allelic variant (i.e., MOI = 1).

**Fig. 2:**
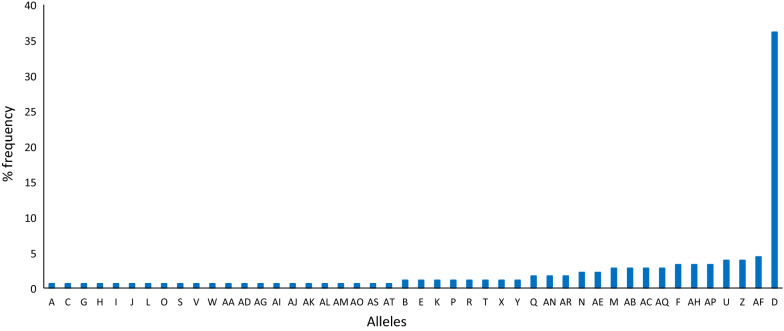
Distribution of the *P. falciparum hrp2* marker allelic variants in 180 isolates from the Tehama region, Yemen

Analyses of these *pfhrp2* sequences showed a high degree of genetic diversity with 46 distinct allelic variants (477–969 bp, corresponding to 159–323 amino acids) found circulating in the area. Of these, 45 alleles (97.8%) occurred in very low frequency (< 5%), though one (540 bp) was dominant as it was found in 65 of the 180 analysed samples (36.1%) (Fig. 2). Details of the *hrp2* allelic variants classification are provided in Additional file 1.

### PCR correction of recurrent cases based on pfhrp2 as a genotyping marker

**Fig. 3:**
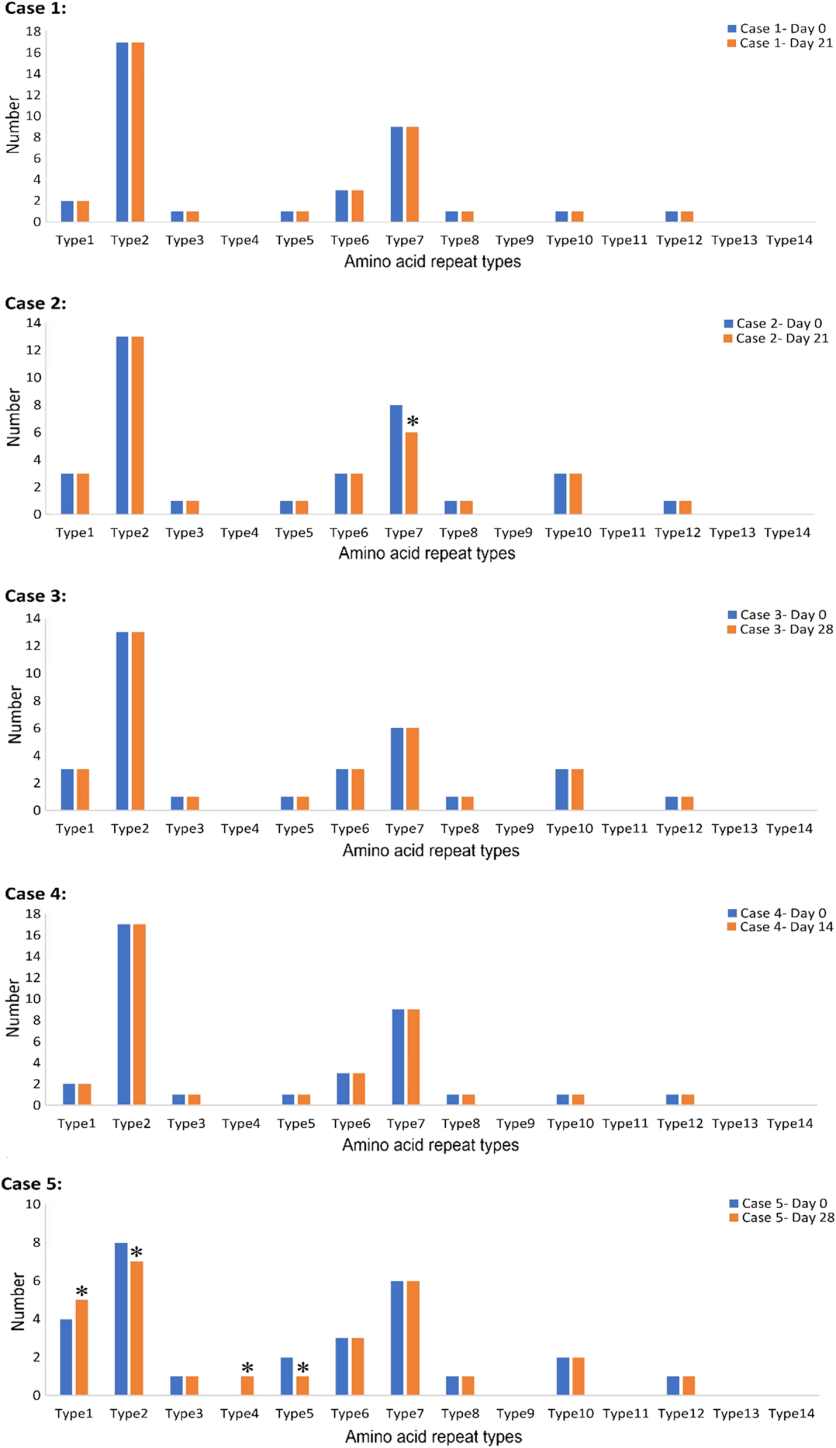
Types and numbers of *pfhrp2* amino acid repeat units for distinguishing between cases of recrudescent or re-infection from a clinical trial in Tehama region, Yemen. * Difference in occurrence, type, or number of amino acid repeat units

Analysis of the paired samples from the five cases with recurrent parasitaemia was conducted by *pfhrp2* amplification followed by sequencing. Comparison of the amino acid type repeats and their frequency in the baseline (day 0) and day X sample pairs, indicated that the outcome for two of the patients could be classed as a re-infection (case 2 and case 5), while the other three cases could be classed as recrudescences, i.e., treatment failures (Fig. 3). These results were entirely consistent with the PCR correction that was carried out previously using the standard *msp1*, *msp2* and *glurp* markers (Table 2) [15].

**Table 2:** Discrimination of recrudescent from new infection episodes from cases in the drug efficacy trial in the Tehama region, Yemen based on *msp1*, *msp2* and *glurp* genotyping

Case	*Msp1*			*Msp2*		*Glurp*	Outcome
	K1	MAD20	RO33	FC27	IC		
Case 1							
Day–0	180	–	–	300	–	1000	RECRUDESCENCE
Day–21	180	–	–	300	–	1000	
Case 2*							
Day–0	–	–	**150**	**450**	–	**900**	NEW INFECTION
Day–21	**200**	–	–	–	**500**	**1000**	
Case 3							
Day–0	180	–	–	–	500	1000	RECRUDESCENCE
Day–28	180	–	–	–	500	1000	
Case 4							
Day–0	180	–	150	300	600	900	RECRUDESCENCE
Day–14	180	–	150	300	600	900	
Case 5*							
Day–0	**200**	–	–	**350**	–	900	NEW INFECTION
Day–28	–	**230**	–	**280**	–	900	

*Cases for which the D0/DX samples genotyping patterns differed are presented in bold

**Fig. 4:**
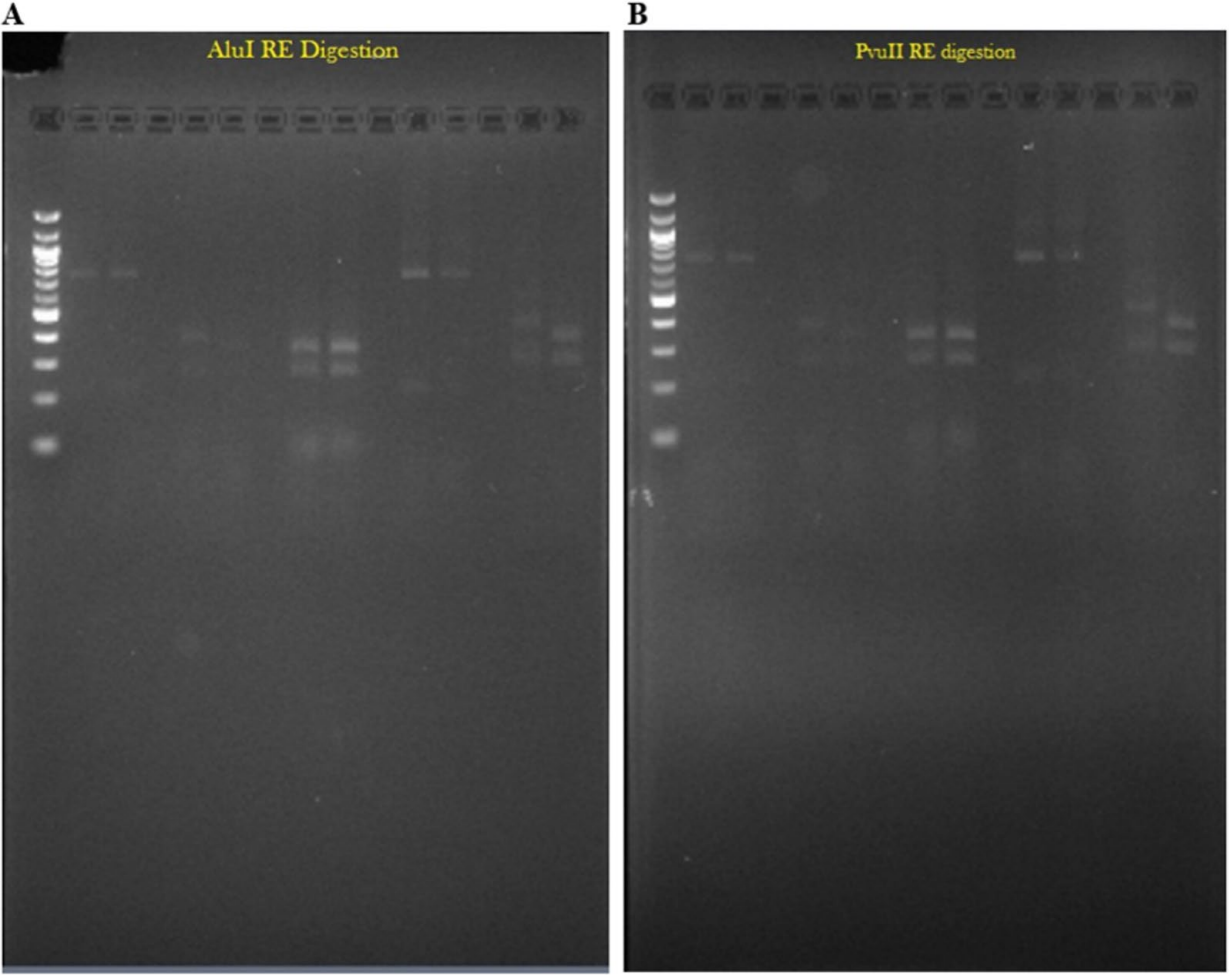
Digestion pattern of *pfhrp2* PCR products using **A** *Pvu* II, and **B** *Alu* I restriction enzymes. Lane 1: 100 bp DNA ladder, Lane 2: case 1, day 0; Lane 3: case 1, day 21, Lane 5: case 2, day 0; Lane 6: case 2, day 21, Lane 8: case 3, day 0; Lane 9: case 3, day 28, Lane 11: case 4, day 0; Lane 12: case 4, day 14, Lane 14: case 5, day 0, Lane 15: case 5, day 28

Two restriction enzymes, *Alu* I or *Pvu* II, have been selected for RFLP analysis, and the digests of the amplified *pfhrp2* fragments from the paired samples from the five cases were compared in order to reveal potential differences between allelic variants without the need for sequencing. The presence of an identical number and size of the resulting bands derived from the day 0 and day X sample pairs indicated a recrudescence (cases 1, 3 and 4), while distinct patterns for the paired samples indicated a re-infection (cases 2 and 5) (Fig. 4A and B). These results were consistent with those based on genotyping based on the analysis of the *pfhrp2* repeats sequences as well as that based on the standard *msp1*, *msp2* and *glurp* genotyping protocols.

## Discussion

Malaria drug efficacy clinical trial in endemic areas remains the gold standard method for monitoring changes in the efficacy of antimalarial drugs, and their outcomes guide the treatment policy adopted nationally. Correction of these outcomes via parasites genotyping, in which treatment failures are distinguished from re-infections that might have arisen during the follow-up period, has substantially improved outcome accuracy. In recent years, three genetic markers with standardised genotyping protocols have been adopted [1] in order to ensure consistency and comparability between studies. The validity of one of these markers (*glurp*) has been recently put into question [5], and in some regions low allelic diversity for the one or other of the other two markers (*msp1* and *msp2*) diminishes their adequacy [18–20]. The likelihood of low genetic diversity increases in areas of low transmission intensity or in those where anti-malarial measures are leading to near elimination. In such cases, additional polymorphic genetic markers will need to be considered to achieve PCR correction of trial outcomes.

Given the very high diversity observed for the variable repeat region of the *pfhrp2* exon in many malaria endemic areas [21, 22], including those with low transmission intensity, its usefulness was assessed as a potential additional genetic marker for PCR correction analyses. Although amplicon size could be sufficient to discriminate between two isolates, two post-amplification strategies with a higher resolution were employed to distinguish between the various allelic variants: amplicon sequencing, an onerous though highly accurate method, and RFLP analysis, a less sensitive but more field-friendly approach. In the present study, the potential of *pfhrp2* as an additional marker for PCR correction analyses in Yemen has been demonstrated. Genotyping of the paired samples from the five cases with recurrent episodes confirmed the classification (recrudescence or re-infection) that was obtained using the three standard markers. Moreover, in this case, this was equally achieved by direct sequencing of the PCR product as well as through restriction enzymes digestion of the amplicons. It must be noted that this preliminary study concerned only 5 cases that were sampled about seven years ago. Therefore, validation of this marker will require further studies to confirm its usefulness.

Nevertheless, it must be stressed that the value of *pfhrp2* as a genotyping marker is severely compromised in areas where this gene is fully or partially deleted in a large proportion of the circulating parasites. *Plasmodium falciparum* parasites lacking *pfhrp2* were first noted in Peru, where 41% of the samples collected proved negative for this gene [23]. Similar observations were made in the following year with samples collected in various South American countries [24–29], though much lower proportions of such parasites were recorded in other areas at that time [30–32]. A recent systematic review on the prevalence of *pfhrp2* negative parasites indicated that their prevalence is highly variable and potentially equally focal [33]. Thus, *pfhrp2* must not be used as a maker for PCR correction in areas where the prevalence of the parasites lacking the exon 2 of this gene exceeds a few percentage points (10% as a conservative suggestion). Nonetheless, given its high genetic diversity with allelic variants that can be distinguished by amplicon length, restriction digestions patterns and eventually sequencing, *pfhrp2* can serve as a first marker to be analysed from paired samples; any cases thereby clearly classed as re-infection could then be excluded from those that would require genotyping by the other markers (*msp1* and *msp2*), thus saving time and resources.

## Conclusion

The polymorphic *pfhrp2* gene has the potential to act as an additional genetic marker for inclusion in the PCR genotyping protocols employed to correct the outcomes of in vivo efficacy drug trials conduction in malaria endemic areas. However, its use is restricted to endemic areas where only a very small minority of *P. falciparum* population lack the *pfhrp2* gene.

## Supplementary Information

**Additional file 1.** Description and classification of the *pfhrp2* allelic variants classification and their frequencies.

## Data Availability

The data that support the findings of this study are available from the corresponding author upon reasonable request.

## Abbreviations

ACT: Artemisinin-based combination therapy
*msp1*: Merozoite surface protein 1
*msp2*: Merozoite surface protein 2
*glurp*: Glutamate-rich protein
*pfhrp2*: *P. falciparum* Histidine-rich protein 2
AS: Artemisinin
SP: Sulfadoxine-pyrimethamine
